# Perceptions and Behaviors Regarding Early Screening and Breast-Related Complaints Among Saudi Women

**DOI:** 10.7759/cureus.61891

**Published:** 2024-06-07

**Authors:** Ashwaq J Aljehani, Mohammed A Alomar, Abdulaziz M Albalawi, Abdullah S Alsultan, Firas O Alhussini, Riyadh F Alshehri, Abdulaziz A Bin Masoud, Fahad S Alshehri

**Affiliations:** 1 Department of Surgery, Imam Mohammad Ibn Saud Islamic University, Riyadh, SAU; 2 College of Medicine, Imam Mohammad Ibn Saud Islamic University, Riyadh, SAU

**Keywords:** screening of breast cancer, awareness of breast cancer, breast self‑examination, breast cancer perception, breast screening, breast cancer

## Abstract

Background

Breast cancer (BC) is a global public health issue, contributing to a significant death toll among women. Breast cancer is the most common type among Saudi women, accounting for over a quarter of all new cancer cases. The current approaches for detecting BC include mammography, clinical breast exams, and breast self-examination (BSE). Early diagnosis of BC is crucial for reducing mortality and morbidity. This study aims to investigate attitudes and behaviors regarding early screening and self-examination of breast cancer in Saudi Arabia.

Materials and method

This cross-sectional study was conducted over eight months. The sample size calculation with a 95% confidence interval and 0.05 precision rate is 600 of the total targeted group. The study included Saudi females aged 30 and above. Data were collected via an online questionnaire. The questionnaire evaluated various aspects, including information regarding sociodemographics, barriers, and attitudes toward breast cancer screening.

Results

The majority of participants were aged 41-50 (40.7%) and married (76.2%). Over a third (31.9%) had been diagnosed with benign breast tumors, with MRI being the most common examination method (39.2%). Regarding screening practices, 55.7% had been screened previously, with a high intention for future screening (76.8%). Attitudes toward screening were positive, with 83.4% willing to undergo testing if free, painless, and conducted by a female provider. Barriers to screening included fear of examination (30.2%) and shame about exposing the body (25.6%).

Conclusion

The study provides valuable insights into the demographic characteristics, prevalence of benign breast tumors, screening practices, and attitudes toward breast cancer screening among Saudi women aged 30 and above. Factors such as age, marital status, income, occupation, and geographical residency influence screening behavior and intentions. Efforts to promote awareness, reduce barriers, and improve access to screening services are essential for enhancing breast cancer detection and prevention within this population.

## Introduction

Breast cancer (BC) is a global public health issue, and it has become the most widespread cancer among women, contributing to a significant death toll among them [1]. Breast cancer remains the most prevalent cancer among Saudi women, accounting for more than 25% of all new cancer cases, according to recent Saudi cancer registry statistics [2]. BC was classified first among women, and there were a total of 1979 female BC cases in 2015. BC accounted for 16.7% of all cancers documented by Saudi citizens and 30.1% of all cancers reported by women of all ages. For the Saudi female population, the age-standardized incidence rate (ASR) per 100,000 people was 24.3. The incidence appears to vary by geographic location, with the highest frequency observed in the country's eastern, central, and western regions [3]. The median age of diagnosis was 50 years. According to a recent study on the impact of BC mortality in Saudi Arabia, fatalities related to BC are anticipated to quadruple between 2025 and 2050 [4]. Mammography, clinical breast examinations, and breast self-examination (BSE) are the current methods for identifying BC [4]. Before 2002, mammography was introduced in the Kingdom of Saudi Arabia [5]. In 2007, a countrywide BC screening facility was created in Riyadh, and 1,215 were screened in the first year [5]. Another local mammography screening program, aimed at women aged 35 to 60, was held in Al Qasim in 2007 and was preceded by an awareness program [6]. While mammography has been offered in all areas of KSA since 2005, the National Saudi Health Interview Survey (SHIS) 2015 found a relatively low incidence of breast cancer screening (BCS), with 1,135 women aged 50 or older reporting that they had not undergone a clinical breast examination (CBE) and 92% had not had a mammogram in the previous year [7]. Early identification of BC is critical for lowering both morbidity and mortality. According to several studies conducted in Saudi Arabia, Saudi women are under-informed about BC and face considerable barriers to making an early presentation [8-10]. Men managing women's decisions and activities is a major sociocultural obstacle to breast screening and early BC detection in many conservative countries. Cultural, ethnic, and legal restraints are a few of the non-economic obstacles hindering early discovery. Even with enough resources, failure to recognize these challenges might jeopardize the success of any cancer treatment program [11]. The purpose of the study is to investigate attitudes and behaviors regarding early screening and self-examination of breast cancer in Saudi Arabia and to determine the factors and obstacles that affect breast cancer-related screening and examination among people in the Saudi population.

## Materials and methods

Study design

The cross-sectional study was conducted over eight months, targeting Saudi women aged 30 years and above. The sample size calculation, with a 95% confidence interval and a precision rate of 0.05, determined that 600 participants were required. The study focused on Saudi females aged 30 and above who had undergone early breast screening, excluding males and females under 30. Data collection was facilitated through an online questionnaire. To ensure the accuracy and validity of the questionnaire, a pilot study involving approximately 15 participants was conducted to confirm the language clarity and question validity. All participants were provided with informed consent, ensuring confidentiality and the sole use of information for scientific research purposes. The ethical approval of the study was obtained by the chairman of the Institutional Review Board (IRB), Prof. Abdulaziz Al-Akaabba, at the Imam Mohammad Ibn Saud Islamic University research ethics committee in Riyadh, Saudi Arabia (reference number: 569/2023; dated: 09-01-2024).

A structured self-response questionnaire was administered to assess various variables, including sociodemographic factors such as age, gender, nationality, marital status, number of children, family income, benign breast lesions, screening methods, screening recommendations, educational level, occupational status, and residential areas. Participants were categorized into three age groups: 30-39, 40-49, and 50 or above, and based on marital status: single, married, divorced/widowed. Residential areas were classified as urban or rural, and the number of children was categorized as less than four or more than four. The questionnaire included a section for univariate analysis focusing on whether participants had ever undergone breast screening (clinical breast examination and mammography), considering variables such as residence (urban or rural), age groups, educational status, working status, marital status, family income, use of hormonal contraceptives, and previous benign lesions. Another section of the questionnaire explored barriers to breast cancer screening, addressing concerns such as discomfort with body touch, embarrassment, lack of awareness about societal perceptions, stigma, cultural taboos, fear of hospitals or doctors, pain during examinations, time constraints, and inadequate awareness programs. Participants were also asked about reasons for not attending breast cancer screening, with options including lack of time, disinterest, logistical challenges, perceived lack of necessity, fear of results, personal/family issues, prior screening, illness, or pregnancy. Furthermore, participants' attitudes toward breast cancer screening were assessed based on agreement or disagreement with statements such as "early breast cancer detection is crucial for prevention" and "I am considering breast cancer screening seriously in the near future." Additional items included a willingness to undergo mammography if it were free, painless, and conducted by a female provider.

Statistical analysis 

Microsoft Excel was used for data entry, cleaning, and coding, while data analysis was performed using the IBM Corp. Released 2019. IBM SPSS Statistics for Windows, Version 26.0. Armonk, NY: IBM Corp. with the assistance of a data analysis expert, ensuring accurate interpretation and presentation of results. Frequency and percentage were used to describe the categorical variables. A chi test and an ANOVA test were used to assess the relation between practice and attitude with demographic variables. All statements were considered significant when the p-value was lower than 0.05. 

## Results

The demographic characteristics of the included participants (n=609) reveal insights into the distribution across various factors. Regarding age distribution, the majority of participants fell within the 41-50 age group (40.7%), followed closely by those aged >50 years (32.2%), while 27.1% were aged 30-40. Marital status showed that the majority were married (76.2%), with single individuals comprising 10.8% and divorced/widowed participants at 13.0%. In terms of family size, a slight majority had four or more children (61.2%), while 38.8% had fewer than four. The age at first pregnancy varied, with 37.2% falling within the 20-25 age range, followed by 22.3% at 20 or younger. Regarding monthly income, the distribution was fairly even across income brackets, with the highest proportion (31.9%) earning between 10,000 and 15,000 SR. Educationally, the majority held a college degree (61.4%), followed by high school (25.3%). In terms of occupation, a significant portion was unemployed (43.5%), with governmental employees comprising 43.0%. Geographically, the central region had the highest representation (56.0%), followed by the southern region (12.6%) (Table 1).

**Table 1:** Demographic characteristics of the included participants (n=609) The data have been presented as count and N (%). SR = Saudi riyal

Demographics		Count	N (%)
Age	30-40	165	27.1%
	41-50	248	40.7%
	> 50 years	196	32.2%
Marital status	Single	66	10.8%
	Married	464	76.2%
	Divorced/Widow	79	13.0%
Number of children	< 4 children	210	38.8%
	4 or more children	331	61.2%
Age at first pregnancy	20 or younger	122	22.3%
	20-25	204	37.2%
	25-30	175	31.9%
	30-35	38	6.9%
	35 or older	9	1.6%
Monthly income	5000 SR or lower	57	9.4%
	5000 - 10000 SR	180	29.6%
	10000 - 15000 SR	194	31.9%
	15000 SR or higher	178	29.2%
Educational level	Primary	10	1.6%
	Intermediate	45	7.4%
	High school	154	25.3%
	College	374	61.4%
	Master	14	2.3%
	Doctoral	12	2.0%
Occupation	Unemployed	265	43.5%
	Governmental employee	262	43.0%
	Private section employee	82	13.5%
Residency	Central region	341	56.0%
	Northern region	52	8.5%
	Southern region	77	12.6%
	Western region	65	10.7%
	Eastern region	74	12.2%

The prevalence of benign breast tumors among participants indicated that 31.9% had been diagnosed with such tumors, while 68.1% had not. Among those diagnosed, the most common examination method was MRI (39.2%), followed by ultrasound (37.6%), mammogram (22.2%), and a combination of all techniques (1.0%). Regarding those who advised participants to undergo examinations, friends (39.7%) and social media (35.6%) were the most prevalent sources of recommendation (Table 2).

**Table 2:** Prevalence of breast tumor The data have been presented as count and N (%). MRI: Magnetic resonance imaging

Diagnosis		Count	N (%)
Have you ever been diagnosed with a benign breast tumor?	No	415	68.1%
	Yes	194	31.9%
Examination method?	Mammogram	43	22.2%
	Ultrasound	73	37.6%
	MRI	76	39.2%
	All these techniques	2	1.0%
Who advised you to do the examination?	Relatives	25	12.9%
	Friend	77	39.7%
	Social media	69	35.6%
	Healthcare providers	6	3.1%
	Personal decision	17	8.8%

Regarding screening practices and attitudes toward breast cancer, 55.7% of participants reported having been screened before, while 44.3% had not. A significant proportion (76.8%) expressed a serious intention to undergo screening in the near future. Additionally, a large majority (83.4%) indicated a willingness to undergo testing if it were free, painless, and conducted by a female provider (Table 3).

**Table 3:** Practice and attitude toward screening for breast cancer The data have been presented as count and N (%).

Breast screening		Count	N (%)
Have you been screened for breast cancer before? Either by examining the breast or by taking a mammogram?	No	270	44.3%
	Yes	339	55.7%
I seriously intend to get screened for breast cancer in the near future:	Disagree	141	23.2%
	Agree	468	76.8%
I will test it if it is free, painless and performed by a woman:	Disagree	101	16.6%
	Agree	508	83.4%

**Figure 1:**
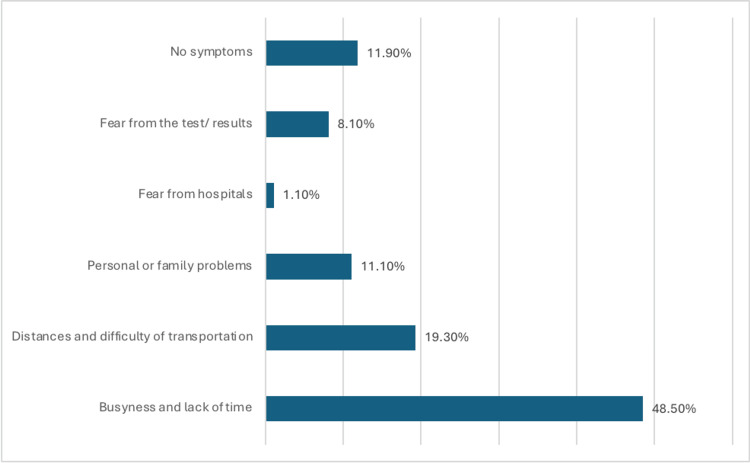
Among participants never screened, why haven't you detected breast cancer yet?

Among participants who had never been screened, the reasons included busyness and lack of time (48.5%), distance and transportation difficulties (19.3%), and personal or family problems (11.1%) (Figure 1).

**Figure 2:**
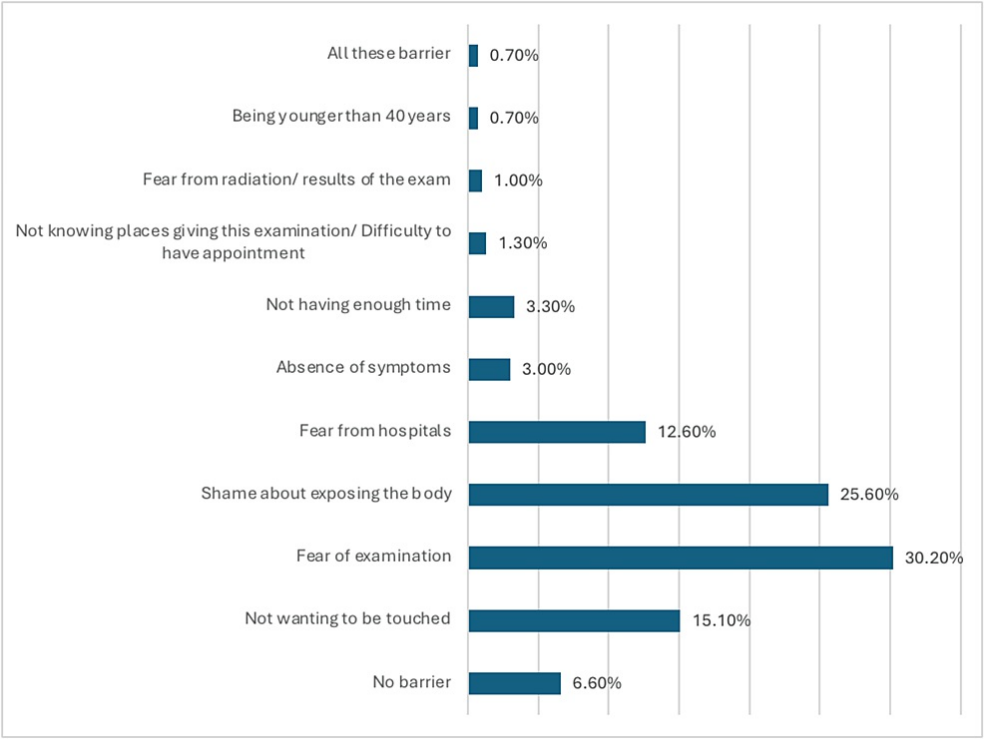
What is the barrier that prevents you from attending clinical examinations to detect breast cancer?

Barriers to attending clinical examinations for breast cancer detection were varied, with fear of examination (30.2%) and shame about exposing the body (25.6%) being the most prevalent (Figure 2).

The analysis of the relationship between participants' practice and attitude toward breast cancer screening and various demographic factors yielded significant insights. Age emerged as a significant factor influencing screening behavior, with statistically significant differences observed among age groups (p=0.000). Participants aged 41-50 and those over 50 years showed higher rates of previous screening compared to those aged 30-40. However, intention for future screening did not significantly differ across age groups. Marital status also exhibited a notable association with screening behavior and intention (p=0.027 and p=0.011, respectively). Divorced or widowed individuals demonstrated higher rates of previous screening compared to single individuals, and they also expressed a stronger intention for future screening. Similarly, participants with lower monthly incomes were more likely to have been screened previously (p=0.007), although income did not significantly impact future screening intentions. Occupation significantly influenced both previous screening behavior and future screening intentions (p=0.663 and p=0.051, respectively). Unemployed participants exhibited higher rates of previous screening compared to governmental and private sector employees. However, governmental employees showed a stronger intention for future screening compared to unemployed individuals. The geographical residency also played a role in screening behavior, with significant differences observed among regions (p=0.000). Participants from the central region exhibited higher rates of previous screening compared to those from the western region. However, future screening intentions did not significantly vary across regions (Table 4).

**Table 4:** The relation between practice and attitude and demographic factors of the participants The data have been presented as count, N (%), and p-value. *p value <0.05 is considered significant. SR = Saudi riyal

Demographics		Have you been screened for breast cancer before? Either by examining the breast or by taking a mammogram.					I seriously intend to get screened for breast cancer in the near future:				
		No		Yes		Overall p-value	Disagree		Agree		Overall p-value
		Count	N (%)	Count	N (%)		Count	N (%)	Count	N (%)	
Age	30-40	97	58.8%	68	41.2%	0.000*	39	23.6%	126	76.4%	0.657
	41-50	97	39.1%	151	60.9%		53	21.4%	195	78.6%	
	> 50 years	76	38.8%	120	61.2%		49	25.0%	147	75.0%	
Marital status	Single	38	57.6%	28	42.4%	0.027*	25	37.9%	41	62.1%	0.011*
	Married	204	44.0%	260	56.0%		99	21.3%	365	78.7%	
	Divorced/Widow	28	35.4%	51	64.6%		17	21.5%	62	78.5%	
Number of children	< 4 children	93	44.3%	117	55.7%	0.463	39	18.6%	171	81.4%	0.330
	4 or more children	136	41.1%	195	58.9%		73	22.1%	258	77.9%	
Monthly income	5000 SR or lower	34	59.6%	23	40.4%	0.007*	20	35.1%	37	64.9%	0.082
	5000 - 10000 SR	67	37.2%	113	62.8%		37	20.6%	143	79.4%	
	10000 - 15000 SR	96	49.5%	98	50.5%		39	20.1%	155	79.9%	
	15000 SR or higher	73	41.0%	105	59.0%		45	25.3%	133	74.7%	
Educational level	Primary	4	40.0%	6	60.0%	0.777	3	30.0%	7	70.0%	0.684
	Intermediate	17	37.8%	28	62.2%		8	17.8%	37	82.2%	
	High school	73	47.4%	81	52.6%		37	24.0%	117	76.0%	
	College	163	43.6%	211	56.4%		84	22.5%	290	77.5%	
	Master	8	57.1%	6	42.9%		5	35.7%	9	64.3%	
	Doctoral	5	41.7%	7	58.3%		4	33.3%	8	66.7%	
Occupation	Unemployed	123	46.4%	142	53.6%	0.663	74	27.9%	191	72.1%	0.051
	Governmental employee	112	42.7%	150	57.3%		50	19.1%	212	80.9%	
	Private section employee	35	42.7%	47	57.3%		17	20.7%	65	79.3%	
Residency	Central region	163	47.8%	178	52.2%	0.000*	88	25.8%	253	74.2%	0.498
	Northern region	18	34.6%	34	65.4%		9	17.3%	43	82.7%	
	Southern region	26	33.8%	51	66.2%		15	19.5%	62	80.5%	
	Western region	41	63.1%	24	36.9%		14	21.5%	51	78.5%	
	Eastern region	22	29.7%	52	70.3%		15	20.3%	59	79.7%	

## Discussion

Breast cancer remains a significant health concern globally, and early detection through regular screening plays a crucial role in reducing mortality rates associated with the disease. This study aimed to investigate the demographic characteristics, prevalence of benign breast tumors, screening practices, and attitudes toward breast cancer screening among Saudi women aged 30 years and above. The findings provide valuable insights into the factors influencing breast cancer detection behaviors within this population.

The prevalence of benign breast tumors among study participants highlights the importance of early detection and regular screening in identifying potentially harmful lesions. The prevalence of benign breast tumors among our participants was 31.9%, which is higher than reported in a previous study conducted in Egypt where the prevalence of benign breast tumors was 20%. [12]. While the majority of participants had not been diagnosed with benign tumors, a significant proportion (55.7%) had undergone screening, indicating proactive health-seeking behaviors. This is similar to what was reported in some previous Saudi studies, including the study of Alenezi A et al. [13] in Aljouf province, who reported that 48.8% of the women underwent mammography, and the study of AlAbdulkader A et al. [14] in Eastern province, who reported a prevalence of 48.9%. However, this is higher than reported in different studies conducted in Saudi Arabia, including studies of Abdel-Aziz S et al. [15] in Al Hassa with a prevalence of 16.2%, Bakarman M et al. [16] in Jeddah with a prevalence of 18.9%, Alshammari S et al. [17] in Riyadh with a prevalence of 18.7%, Al-Zalabani A et al. [10] in Madinah with a prevalence of 27.7%, and Al-Wassia R et al. [18] in different five regions in Saudi Arabia with a prevalence of 40.2%, and lower than the results of Heena H et al. [19] in Riyadh with a prevalence of 70.2%. This finding underscores the importance of promoting awareness and education about breast health and the benefits of regular screening, even in the absence of symptoms [20].

Barriers to attending clinical examinations for breast cancer detection were also identified, with fear of examination and shame about exposing the body being the most prevalent. These barriers reflect psychological and cultural factors that may deter individuals from seeking healthcare services, underscoring the need for targeted interventions to address misconceptions and alleviate fears surrounding breast cancer screening [21]. This is similar to some previous studies conducted in Saudi Arabia, including the study of Abdel Aziz S et al. [15], who reported that personal anxieties (particularly fear of doctors/examiners, fear of hospitals and health facilities, and concern of consequences/results) were identified as the key factors preventing women from using the free BC screening.

Age emerged as a significant factor influencing screening behavior, with older participants exhibiting higher rates of previous screening. This finding is consistent with studies showing that older age is associated with increased awareness of breast cancer risk and higher rates of participation in screening programs [22-24]. However, younger women may perceive themselves as being at lower risk and may therefore be less likely to engage in screening behaviors [25,26].

Marital status also played a role in screening behavior, with divorced or widowed individuals demonstrating higher rates of previous screening compared to single individuals. This finding is consistent with research suggesting that social support networks, which may be more prevalent among married or previously married individuals, can influence health-related behaviors, including screening uptake [27]. Moreover, divorced or widowed individuals may have experienced health scares or concerns that prompted them to prioritize their health through regular screening.

Income and occupation were also associated with screening behavior and intentions. Participants with lower monthly incomes were more likely to have been screened previously, while governmental employees exhibited a stronger intention for future screening. These findings highlight the influence of socioeconomic factors on access to healthcare services, including screening programs. Individuals with lower incomes may have greater access to subsidized healthcare services or community-based screening initiatives, whereas those with higher incomes may face barriers related to time constraints or perceptions of low risk [28].

Geographical residency emerged as another significant factor influencing screening behavior, with participants from the central region exhibiting higher rates of previous screening. This finding may reflect differences in healthcare infrastructure and access to screening facilities across regions. Urban areas, typically more developed, may have better access to healthcare services and higher levels of health literacy, leading to increased screening uptake [29]. Conversely, rural or remote areas may face challenges related to healthcare access, including limited screening facilities and transportation barriers.

The attitudes toward breast cancer screening revealed a positive inclination among participants, with the majority expressing a serious intention to undergo screening in the near future. Additionally, a high proportion indicated a willingness to undergo testing if it were free, painless, and conducted by a female provider. These findings suggest a favorable attitude toward screening and highlight the importance of addressing practical barriers, such as cost and discomfort, to further improve screening uptake [30].

The study's findings have several implications for healthcare policy and practice in Saudi Arabia. First, efforts to promote breast cancer awareness and education should target younger age groups and single individuals to increase screening uptake among these populations. Second, interventions aimed at reducing barriers to screening, such as providing free or subsidized screening services and addressing cultural sensitivities, can help improve access and participation. Third, healthcare infrastructure and resources should be optimized to ensure equitable access to screening facilities across different regions.

Limitations

Several notable limitations should be considered when interpreting the study findings and designing future research to address the gaps in this area. First, the cross-sectional design provides a snapshot in time and cannot establish causal relationships or track changes over time. Additionally, the use of an online questionnaire may have introduced selection bias, and the absence of qualitative insights restricts the depth of understanding. Also, the study lacks analysis on the influence of a familial history of breast cancer or benign breast lesions on participants' attitudes. Prior research indicates their family's medical history related to breast health can shape their screening practices and perceptions. Without examining this potential factor, the study may have missed an important contributor to women's breast cancer prevention behaviors in Saudi Arabia. Furthermore, reliance on self-reported data may introduce recall bias and social desirability bias, and the sampling may not be fully representative, as the study had a higher proportion from the central region. Finally, the lack of clinical data and limited exploration of barriers to breast cancer screening are also limitations, as this information could provide a more comprehensive understanding of screening behaviors.

## Conclusions

In conclusion, this study provides valuable insights into the demographic characteristics, prevalence of benign breast tumors, screening practices, and attitudes toward breast cancer screening among Saudi women aged 30 years and above. The findings underscore the importance of addressing socioeconomic, cultural, and geographical factors in promoting early detection and improving breast cancer outcomes within this population.
